# Guide and keys for the identification of Syllidae (Annelida, Phyllodocida) from the British Isles (reported and expected species)

**DOI:** 10.3897/zookeys.488.9061

**Published:** 2015-03-19

**Authors:** Guillermo San Martín, Tim M. Worsfold

**Affiliations:** 1 Departamento de Biología (Zoología), Laboratorio de Biología Marina e Invertebrados, Facultad de Ciencias, Universidad Autónoma de Madrid, Canto Blanco, 28049 Madrid, Spain; 2 APEM Limited, Diamond Centre, Unit 7, Works Road, Letchworth Garden City, Hertfordshire SG6 1LW, UK

**Keywords:** Identification, keys, NE Atlantic Ocean, Polychaeta

## Abstract

In November 2012, a workshop was carried out on the taxonomy and systematics of the family Syllidae (Annelida: Phyllodocida) at the Dove Marine Laboratory, Cullercoats, Tynemouth, UK for the National Marine Biological Analytical Quality Control (NMBAQC) Scheme. Illustrated keys for subfamilies, genera and species found in British and Irish waters were provided for participants from the major national agencies and consultancies involved in benthic sample processing. After the workshop, we prepared updates to these keys, to include some additional species provided by participants, and some species reported from nearby areas. In this paper, we provide the revised keys to enable rapid identification of Syllidae from the seas around Britain and Ireland. One new combination, *Palposyllis propeweismanni*, is proposed.

## Introduction

Syllids are small to medium-sized polychaetes (from 2–3 mm long and 15–30 chaetigers, up to about 140 mm and 200 chaetigers). They are extremely abundant and diverse in benthic marine shallow habitats and also inhabit deep areas; however, they are absent from fresh water and are not an important group in estuaries. They are very common on hard substrata, having an errant life among algae, biogenic structures, crevices, within porous rocks, etc. and they also inhabit marine sediments, especially coarse sand, where most species have an interstitial lifestyle. Also, numerous species are associated with other marine organisms, especially sponges and octocorals, mostly in tropical waters.

As syllids may constitute more than 50% (sometimes more than 70%) of the polychaete species that live in some substrata, they are very important in benthic studies. However, because of their small size, they are often overlooked, since most benthic ecology studies are devoted to macrofauna. Furthermore, they are difficult to identify because of their small size and the lack of taxonomic studies and monographs with keys and detailed descriptions for many areas. Syllids are very easy to recognize to family level, because they have a conspicuous modification of the gut, the proventriculus (=proventricle), which constitutes the autapomorphy of the family. The taxonomy and systematics are also complex and difficult, again because of their small size, numerous taxa (approximately 74 genera and 700 species), and the difficulty to correctly observe the characters. This paper is directed to participants of the NMBAQC Scheme and to all laboratory staff and students who need to familiarize themselves with the syllid fauna that may be found in benthic studies from British or Irish waters. Since the workshop, the keys have been modified and completed with the species identified during the workshop, many of which are not yet formally reported in the area. We have included reference numbers (in brackets after each species) to recommended descriptions cited in the references. Comparison of specimens with descriptions and figures is highly recommended. Also, it is necessary to note that fixed specimens lose their pigmentation after some time, and also that young, small specimens have appendages proportionally shorter than large, mature specimens. Also, note that the taxonomy and systematics are not yet completed and some changes and additions are probable in future years. Some genera need careful revision, and some species are only tentatively included in a particular genus, since they do not fit perfectly with the diagnosis of that genus.

Howson and Picton (1997) listed the following species as likely to be found in British water, which are herein arranged according to recent classifications (Aguado and San Martín 2009; Aguado et al. 2007, 2012; Nygren 2004; San Martín and Aguado 2014):

Subfamily **Anoplosyllinae** Aguado & San Martín, 2009: *Streptosyllis bidentata* Southern, 1914; *Streptosyllis websteri* Southern, 1914; *Syllides benedicti* Banse, 1971; *Syllides longocirrata* Ørsted, 1845.

Subfamily **Eusyllinae** Malaquin, 1893: *Eusyllis assimilis* Marenzeller, 1875; *Eusyllis blomstrandi* Malmgren, 1867; *Eusyllis lamelligera* Marion & Bobretzky, 1875; *Nudisyllis divaricata* (Keferstein, 1862); *Nudisyllis pulligera* (Krohn, 1852); *Odontosyllis ctenostoma* Claparède, 1868; *Odontosyllis fulgurans* (Audouin & Milne-Edwards, 1833); *Odontosyllis gibba* Claparède, 1863; *Opisthodonta longocirrata* (Saint-Joseph, 1886); *Pionosyllis compacta* Malmgren, 1867; *Synmerosyllis lamelligera* (Saint-Joseph, 1886).

Subfamily **Exogoninae** Langerhans, 1879: *Brania pusilla* (Dujardin, 1851); *Erinaceusyllis erinaceus* (Claparède, 1863); *Salvatoria clavata* (Claparède, 1863); *Salvatoria limbata* (Claparède, 1868); *Salvatoria swedmarki* (Gidholm, 1962); *Exogone dispar* (Webster, 1879); *Exogone naidina* Ørsted, 1845; *Exogone verugera* (Claparède, 1868); *Parexogone longicirris* (Webster & Benedict, 1887); *Parexogone hebes* (Webster & Benedict, 1884); *Prosphaerosyllis tetralix* (Eliason, 1920); *Sphaerosyllis bulbosa* Southern, 1914; *Sphaerosyllis hystrix* Claparède, 1863; *Sphaerosyllis pirifera* Claparède, 1868; *Sphaerosyllis taylori* Perkins, 1980.

Subfamily **Syllinae** Grube, 1850: *Eurysyllis tuberculata* Ehlers, 1864; *Haplosyllis spongicola* (Grube, 1855); *Syllis amica* Quatrefages, 1866; *Syllis armillaris* (O.F. Müller, 1771); *Syllis cornuta* Rathke, 1843; *Syllis gracilis* Grube, 1840; *Syllis garciai* (Campoy, 1981); *Syllis hyalina* Grube, 1863; *Syllis krohnii* Ehlers, 1864; *Syllis prolifera* Krohn, 1852; *Syllis variegata* Grube, 1860; *Syllis vittata* Grube, 1840; *Trypanosyllis coeliaca* Claparède, 1868; *Trypanosyllis zebra* (Grube, 1860).

Subfamily **Autolytinae** Langerhans, 1879: *Epigamia alexandri* (Malmgren, 1867); *Myrianida brachycephala* (Marenzeller, 1874); *Myrianida edwarsi* (Saint-Joseph, 1886); *Myrianida inermis* (Saint-Joseph, 1886); *Myrianida langerhansi* (Gidholm, 1967); *Myrianida pinnigera* (Montagu, 1808); *Myrianida prolifera* (O.F. Müller, 1788); *Myrianida quinquedecimdentata* (Langerhans, 1884); *Myrianida rubropunctata* (Grube, 1860); *Proceraea aurantiaca* Claparède, 1868; *Proceraea cornuta* (Agassiz, 1862); *Proceraea picta* Ehlers, 1864; *Proceraea prismatica* (O.F. Müller, 1776); *Procerastea halleziana* Malaquin, 1893; *Procerastea nematodes* Langerhans, 1884.

***Incertae sedis*:***Amblyosyllis formosa* (Claparède, 1863); *Dioplosyllis cirrosa* Gidholm, 1962; *Palposyllis prosostoma* Hartmann-Schröder, 1977; *Paraehlersia ferrugina* (Langerhans, 1881); *Streptodonta pterochaeta* (Southern, 1914).

Another 18 syllid taxa were also reported, but they are synonyms of other species, invalid, or doubtful species, or even not recognized as Syllidae.

This number of species is quite low for such an area and it is certain that many other species live in British waters. In the keys below, we have included all previously reported species (excluding invalid or doubtful ones) plus those that have been reported from nearby areas of the NE Atlantic and that could be also present in the study area. Some of these were noted at the NMBAQC workshop or since that time but are not yet formally recorded. It is important to remember the possibility that other species, not in the keys presented here, may yet be found in the area and reference should be made to additional literature for any specimens that do not fit descriptions. Books with keys for syllids of nearby areas include those by Fauvel (1923) (France), Hartmann-Schröder (1996) (Germany), and San Martín (2003) (Iberian Peninsula). A previous NMBAQC workshop (2006) included work on syllids led by Peter Garwood but the resulting key was not published or circulated via the website. Recently, Dietrich et al. (in press) revised the Autolytinae from the area (North Sea and NE Atlantic). Their results are followed here; we strongly recommend use of these keys as a complement to ours for that subfamily.

## Main morphological characters

**Body**

**Figure 1.**
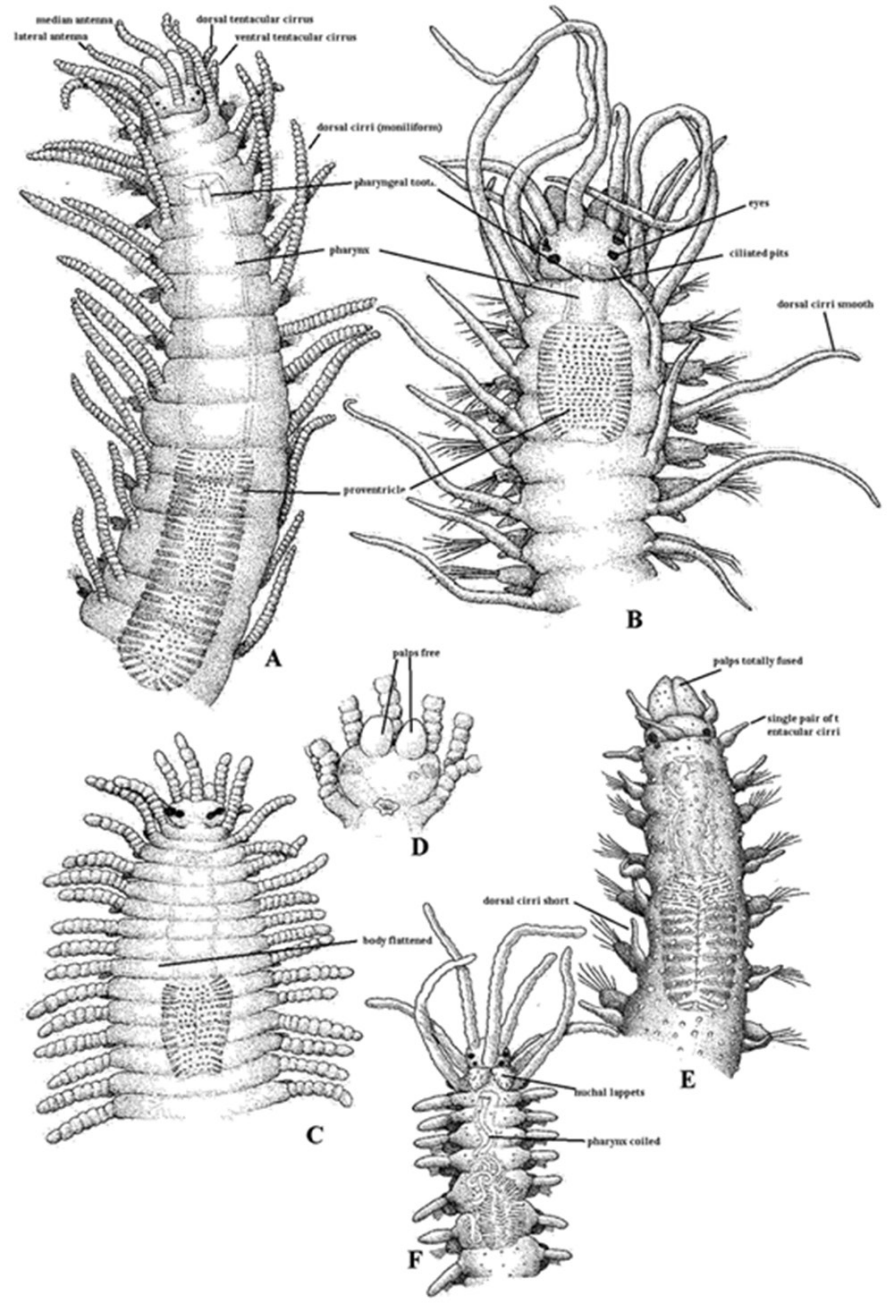
Anterior end of: **A** *Syllis amica* (SF. Syllinae), body cylindrical, smooth surface, two pairs of tentacular cirri, antennae, tentacular and dorsal cirri moniliform, nuchal organs as ciliated pits, palps basally fused **B** *Nudisyllis pulligera* (SF. Eusyllinae), body cylindrical, smooth surface, two pairs of tentacular cirri, antennae, tentacular and dorsal cirri smooth, nuchal organs as ciliated pits, palps free **C** *Trypanosyllis coeliaca* (SF. Syllinae), body flattened, smooth surface, two pairs of tentacular cirri, antennae, tentacular and dorsal cirri moniliform, nuchal organs as ciliated pits, palps free (see figure **D**) **D** Same species, prostomium in ventral view **E** *Sphaerosyllis pirifera* (SF. Exogoninae), body cylindrical, papillated surface, single pair of tentacular cirri, antennae, tentacular and dorsal cirri smooth and short, nuchal organs as ciliated pits, palps totally fused **F** *Myrianida convoluta* (SF. Autolytinae), body cylindrical, smooth surface, two pairs of tentacular cirri, antennae, tentacular and dorsal cirri smooth, nuchal lappets, palps totally fused, pharynx coiled.

Cylindrical in section (Fig. 1A, B, E, F), but may be flattened, ribbon-like (Fig. 1C). The surface is smooth (Fig. 1A–C, F), but may also bear papillae on the dorsal (Fig. 1E) and ventral surface, and on the parapodia. Some bear rugosities, tubercles, rows of cilia, etc.

**Prostomium**

Semicircular to pentagonal or oval and has four eyes and, sometimes, also a pair of ocular spots, three antennae, which may be smooth (Fig. 1B, E, F) or articulated (also known as moniliform) (Fig. 1A, C), short or long, and one pair of palps, triangular in shape, rounded or oval, that may be fully separated from each other (Fig. 1D), basally fused or fused along their entire length (Fig. 1E).

**Tentacular (= peristomial) cirri**

Usually two pairs (Fig. 1A–D, F), but in some genera only one pair (Fig. 1E), or absent, which may be smooth (Fig. 1B, E, F) or articulated (moniliform) (Fig. 1A, C, D), short or long.

**Nuchal organs**

Most commonly as ciliated pits (the most common) but also as nuchal lappets (nuchal epaulettes) (Fig. 1F).

**Parapodia**

**Figure 2.**
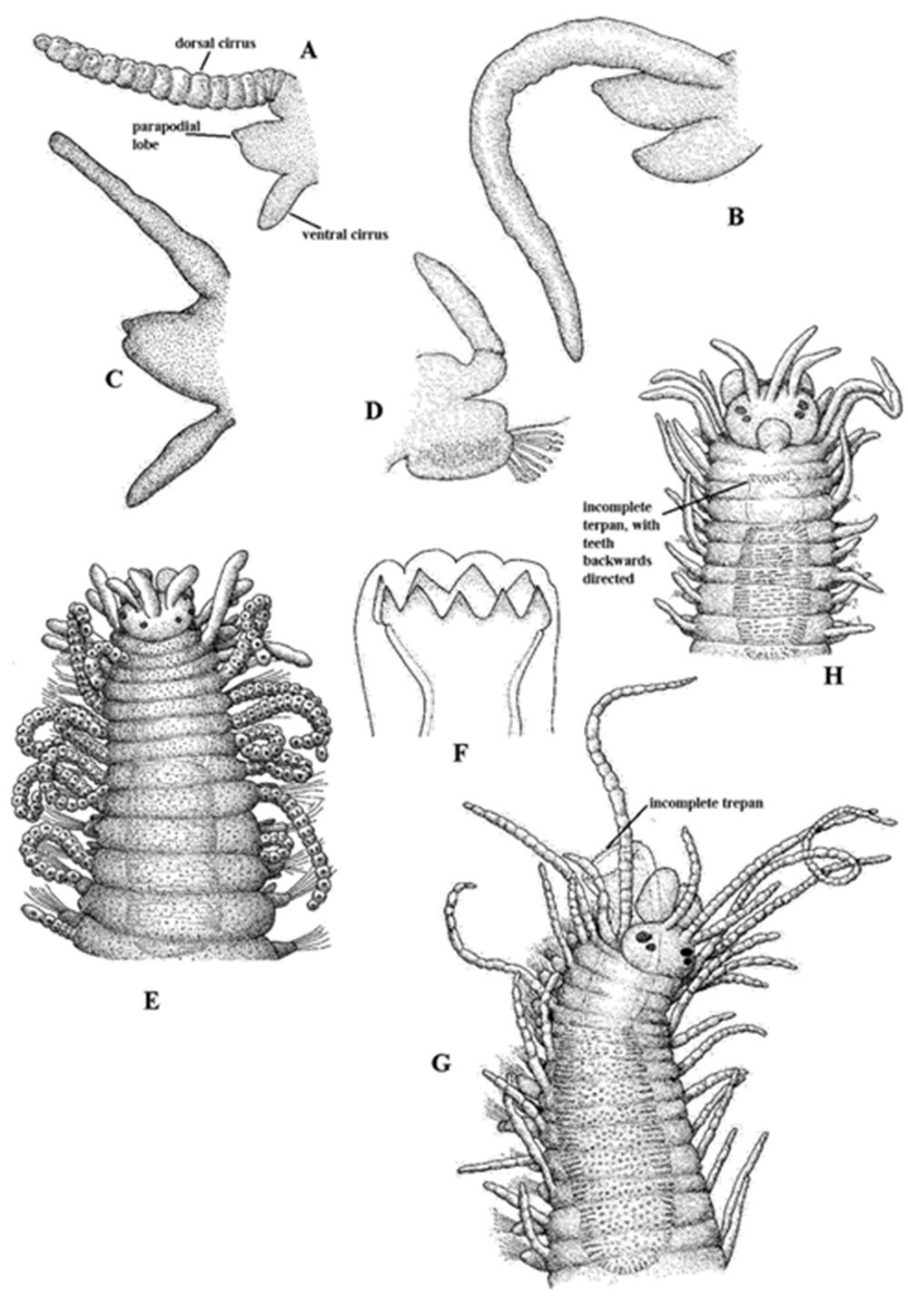
Lateral view of parapodia of: **A** *Syllis amica*, dorsal cirrus moniliform and long **B** *Nudisyllis pulligera*, dorsal cirrus smooth and long **C** *Parapionosyllis brevicirra* (SF. Exogoninae), dorsal cirrus smooth and short **D** *Epigamia labordai* (SF. Autolytinae), dorsal cirrus smooth, short, without ventral cirrus **E** anterior end, dorsal view of *Syllides fulvus* (SF. Anoplosyllinae), without any pharyngeal armature **F** trepan, without middorsal tooth of *Myrianida convoluta* (SF. Autolytinae) **G** everted pharynx of *Eusyllis assimilis* (SF. Eusyllinae), showing an incomplete trepan and middorsal tooth **H** anterior end of *Odontosyllis fulgurans* (SF. Eusyllinae), with an incomplete trepan, teeth directed to posterior part of body.

Uniramous (except on some segments, during reproduction), with dorsal cirri, parapodial lobes, ventral cirri, chaetae, and aciculae (Fig. 2A–D).

**Dorsal cirri**

May be long or short, alternating between long and short, smooth (Figs 1B, E, F, 2B–D) or moniliform (Figs 1A, C, 2A). Typically filiform, but may be of different shapes.

**Ventral cirri**

Present, except in the subfamily Autolytinae, in which they appear to be absent (Fig. 2D) but are in fact fused to parapodial lobes.

**Pharynx**

Usually straight, but coiled in some genera, sometimes very slender and complex (Fig. 1F).

**Pharyngeal armature**

Absent in the subfamily Anoplosyllinae (Fig. 2E), but most often as a single pharyngeal tooth, or as a crown of denticles on the pharyngeal opening, i.e. the trepan, with (Fig. 2G) or without a pharyngeal tooth (Fig. 2F). The trepan may be complete or incomplete, and the denticles may be directed to the anterior or posterior parts of the body (Fig. 2H).

**Proventriculus (= Proventricle)**

Rectangular, squared or barrel-shaped. Size (number of segments) and number of muscle cell rows vary between species.

**Chaetae**

**Figure 3.**
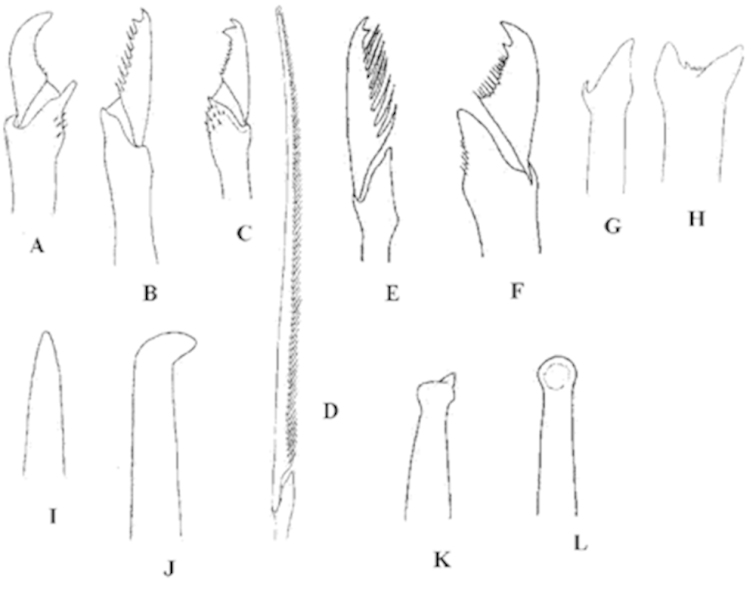
Compound chaetae of **A** *Sphaerosyllis pirifera* (falciger, unidentate, almost smooth on margin) **B** *Trypanosyllis coeliaca* (falciger, bidentate with both teeth similar, moderate spines on margin) **C** *Eusyllis assimilis* (falciger, bidentate, proximal tooth longer than distal one, short spines) **D** *Syllis garciai* (spiniger-like, long spines on margin); **E** *Syllis garciai* (falciger, bidentate, both teeth similar, long spines) **F** *Syllis krohnii* (falciger, bidentate, proximal tooth shorter than distal one, short spines on margin) **G** *Syllis amica* (thick simple chaeta by blade loss and shaft enlargement) **H** *Syllis gracilis* (thick simple chaeta by blade and shaft fusion). Aciculae of: **I** *Trypanosyllis coeliaca* (straight, pointed) **J** *Eusyllis assimilis* (distally bent at an angle) **K** *Syllis gracilis* (acuminate) **L** *Syllis prolifera* (distally rounded).

Typically, compound heterogomph, with capillary dorsal and ventral simple chaetae on posterior parapodia but many modifications may occur. Some may be elongated and similar to the spinigers of nereidids, known as spiniger-like (Fig. 3D), or pseudospingers. Falcigers usually bidentate, with both teeth similar (Fig. 3B), the proximal teeth either smaller than the distal (Fig. 3F) or larger (Fig. 3C). Blades may also be unidentate (Fig. 3A). The blades may be smooth, or have a row of marginal spines, which may be long (Fig. 3E) or short (Fig. 3C, F).

Sometimes, there may be thick simple chaetae due to the loss of blades and enlargement of shafts (Fig. 3G) or by fusion of blade and shaft (Fig. 3H). The capillary dorsal and ventral simple chaetae are usually very slender, bifid or entire, with or without subdistal spines. Typically, these capillary simple chaetae are present only on posterior parapodia.

**Aciculae**

Numerous different kinds of tips may be present: straight and pointed (Fig. 3I), acuminate (Fig. 3K), bent at an angle (Fig. 3J), distally rounded (Fig. 3L), and other variations.

**Reproduction**

There are two main reproductive strategies in syllids: Epigamy and Schizogamy.

**Figure 4.**
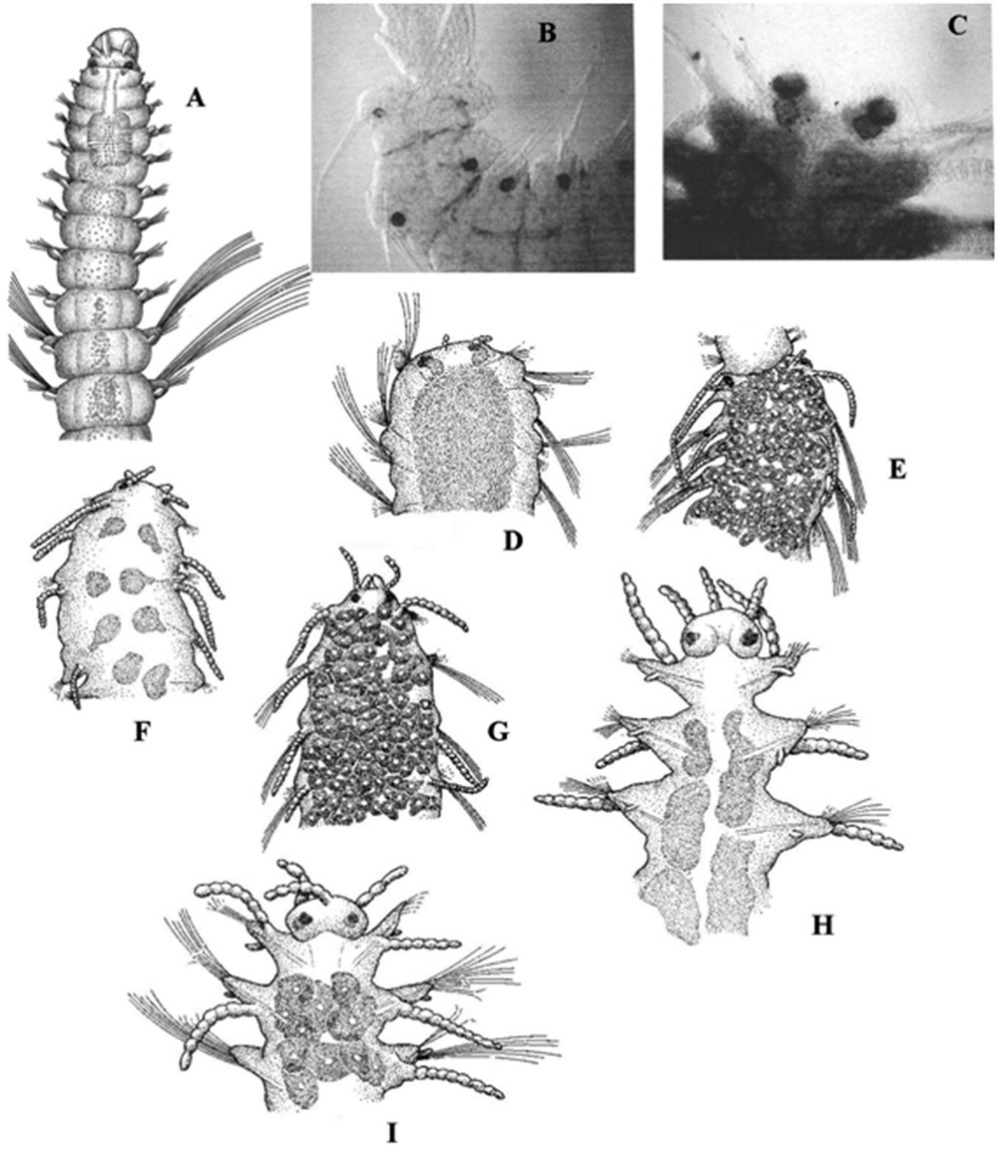
**A** epigamic male of *Exogone naidina*. Anterior end of stolons **B** acephalous (male, still attached to parental), *Haplosyllis spongicola* **C** acerous (male), *Trypanosyllis zebra* **D** dicerous (male), *Syllis prolifera* **E** dicerous (female, still attached to parental), *Syllis prolifera* **F** tetracerous (male), *Syllis pulvinata* **G** tetracerous (female), *Syllis pulvinata* **H** pentacerous (male), *Syllis hyalina* **I** pentacerous (female), *Syllis hyalina*. All, dorsal view, except **H** ventral view.

Epigamy in syllids is quite similar to that of other polychaetes but long, slender notochaetae appear for swimming (natatory chaetae) (Fig. 4A) in some parapodia (from the mid-body backwards). There are two kinds of epigamy: without brooding or with brooding eggs. Brooding eggs may be dorsal (attached by capillary notochaetae) or ventral (attached to nephridial openings). In the latter, juveniles grow attached to mother’s body.

**Figure 5.**
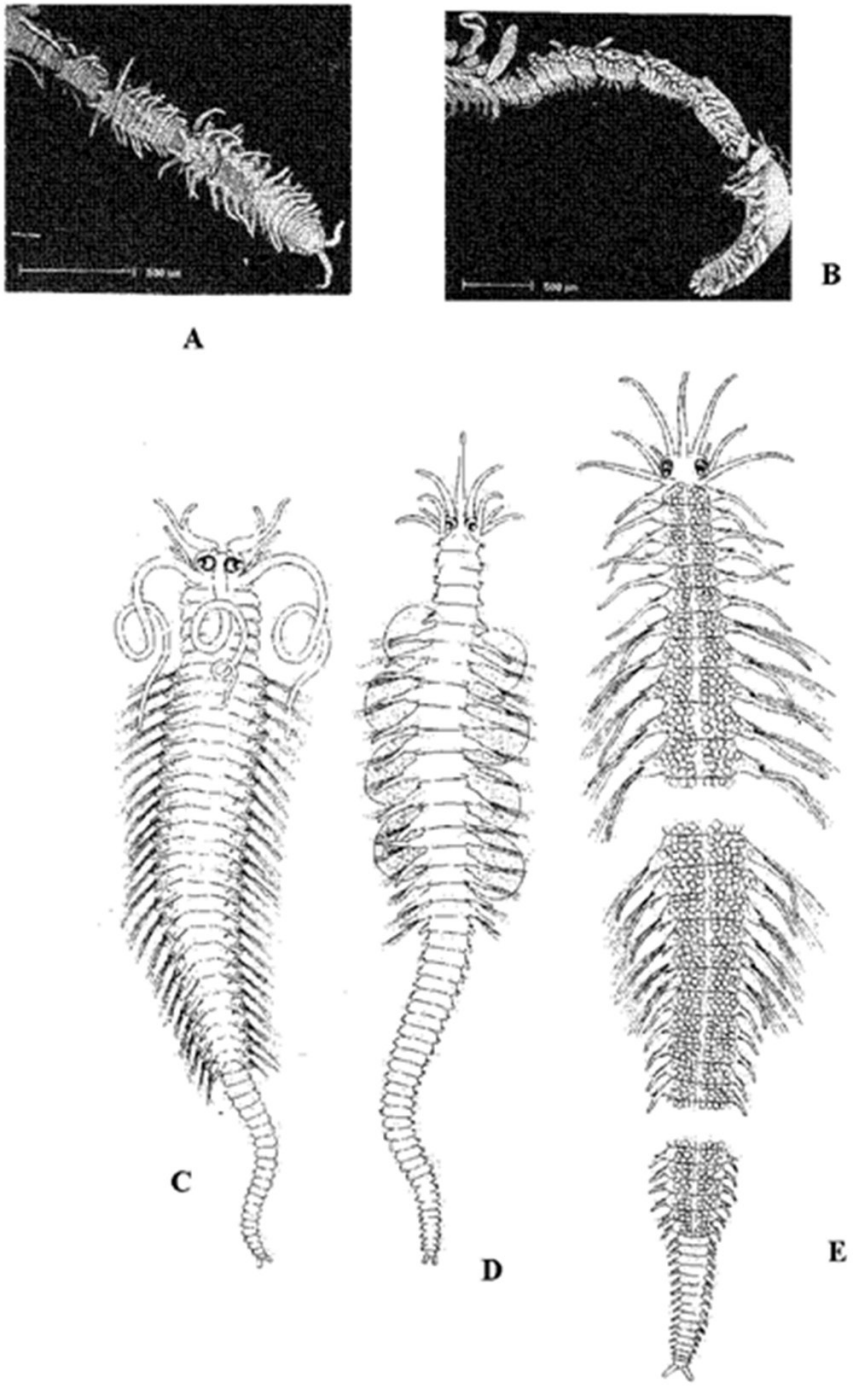
**A, B** Chain of stolons (*Myrianida* spp.) **C** *Polybostrichus* **D** *Sacconereis*, with brooding ventral sac **E** *Sacconereis*.

Schizogamy by means of sexual stolons. Stolons are detached individuals budded off from the adult, without gut, composed of few segments, filled with gametes: a short life, purely for reproduction. There are two kinds of schizogamy: scissiparity (formation of a single stolon) and gemmiparity (formation of a chain of stolons) (Fig. 5A, B).

Stolons of the Syllinae have no sexual dimorphism, but are easily distinguished because males store spermatozoa and females store oocytes; there are different kinds of stolons: acephalous (without ‘head’) (Fig. 4B), acerous (=‘*Tetraglene*’) (a ‘head’ without appendages, and with two pairs of eyes) (Fig. 4C), dicerous (= ‘*Chaetosyllis*’) (a bilobed ‘head’ with two pairs of eyes and two antennae) (Fig. 4D, E), tetracerous (a ‘head’ with two palps and two antennae) (Fig. 4F, G), pentacerous (=‘*Ioida*’) (a ‘head’ with two pairs of eyes, three antennae, and two palps) (Fig. 4H, I).

Stolons of the Autolytinae have marked sexual dimorphism. Male stolons (‘*Polybostrichus*’) have a ‘head’ with two pairs of eyes, two bifid, elongated palps and three antennae, the median one long and spiral (Fig. 5C). Female stolons (‘*Sacconereis*’) have a ‘head’ with two pairs of eyes, two short, simple palps, and three antennae (Fig. 5D, E). Both also have two pairs of ‘tentacular cirri’.

Viviparity has also been reported in some species.

## Key to subfamilies and ‘incertae sedis’ genera

**Table:** 

1	Nuchal organs as two occipital lappets. Pharynx more or less sinuous and coiled	**2**
–	Nuchal organs as two ciliated pits (difficult to observe; occipital lappets absent or, if present, with transversal ridges) between prostomium and peristomium. Pharynx straight	**4**
2	Body composed of few, rhomboidal segments. Ventral cirri well developed. Last segment without chaetae, with two pairs of long cirri	***Amblyosyllis***
–	Body composed of numerous, cylindrical segments. Ventral cirri absent or fused to parapodial lobes. All segments (except peristomium) chaetigerous	**3**
3	Two antennae. Ventral cirri distinct, fused along ventral side of parapodial lobes. Compound chaetae with long, filiform, unidentate blades. Reproduction unknown	***Acritagasyllis***
–	Three antennae. Ventral cirri apparently absent (totally fused with parapodial lobes?). Compound chaetae with short blades, usually with proximal tooth longer than distal. Reproduction by epigamy or schizogamy	**Subfamily Autolytinae**
4	Pharynx unarmed	**5**
–	Pharynx with mid-dorsal tooth, trepan or both	**6**
5	Palps fused along their entire length. Antennae and tentacular cirri minute, papilliform. Single pair of tentacular cirri. Reproduction by epigamy	***Anguillosyllis***
–	Palps fused basally. Antennae and tentacular cirri more or less club-shaped. Two pairs of tentacular cirri. Reproduction by epigamy or brooding eggs ventrally	**Subfamily Anoplosyllinae**
6	Antennae, tentacular cirri and dorsal cirri distinctly articulated, usually long (two genera with only one spherical article). Reproduction by schizogamy (some viviparous)	**Subfamily Syllinae**
–	Appendages smooth or weakly articulated on anterior part of body. Reproduction by epigamy (but unknown in several genera)	**7**
7	Palps fused entirely or at least to mid way along their length. Antennae, tentacular cirri and dorsal cirri short (sometimes papilliform). Eggs brooded dorsally on capillary notochaetae, or ventrally, attached to nephridial pores	**Subfamily Exogoninae**
–	Palps not completely fused. Appendages long, filiform. No brooding of eggs; reproduction by epigamy (or unknown)	**Subfamily Eusyllinae** (plus some *incertae sedis* genera)

### Genus *Amblyosyllis* Grube, 1857

**Table:** 

1	Nuchal lappets short, more or less spherical. Trepan with 6 pentacuspid teeth	***Amblyosyllis madeirensis* Langerhans, 1879** (1)
–	Nuchal lappets long, reaching the level of chaetiger 2. Teeth of trepan otherwise	**2**
2	Trepan with 6 monocuspid teeth, each with a basal spine on each side, more or less developed on larger specimens	***Amblyosyllis formosa* (Claparède, 1863)** (1)
–	Trepan with 6 teeth, each with 11 cusps	***Amblyosyllis finmarchica* (Malmgren, 1867)** (2)

### Genus *Acritagasyllis* Lucas, San Martín & Sikorski, 2010

*Acritagasyllis longichaetosa* Lucas, San Martín & Sikorski, 2010 (3)

### Genus *Anguillosyllis* Day, 1963

*Anguillosyllis pupa* (Hartman, 1965) (4)

### Key to genera of Anoplosyllinae Aguado & San Martín, 2009

**Table:** 

1	Aciculae of some anterior parapodia enlarged, with inflated tips (one exception)	***Streptosyllis***
–	Aciculae unmodified, without inflated tips	**2**
2	Dorsal cirri all smooth, more or less club-shaped	***Anoplosyllis***
–	Dorsal cirri from chaetiger 3 distinctly annulated	***Syllides***

#### Genus *Streptosyllis* Webster & Benedict, 1884

**Table:** 

1	Aciculae not enlarged. Blades of compound chaetae with distinct hoods	***Streptosyllis nunezi* Faulwetter, Vasileiadou, Papageorgiou & Arvanatidis, 2008** (18)
–	Aciculae of some anterior segments enlarged. Blades of compound chaetae without hoods	**2**
2	Compound chaetae with indistinctly bidentate blades. Enlarged aciculae in chaetigers 2–5	***Streptosyllis websteri* Southern, 1914** (1)
–	Compound chaetae with distinctly bidentate blades. Enlarged aciculae in chaetigers 2–6	**3**
3	Blades of compound chaetae with both teeth similar and close to each other. Aciculae of chaetiger 7 only slightly more slender than those of chaetiger 6	***Streptosyllis bidentata* Southern, 1914** (1)
–	Blades of compound chaetae with proximal teeth longer and well separated. Aciculae of chaetiger 7 distinctly more slender than those of chaetiger 6	***Streptosyllis campoyi* Brito, Núñez & San Martín, 2000** (1)

#### Genus *Anoplosyllis* Claparède, 1868

*Anoplosyllis edentula* Claparède, 1868 (1)

#### Genus *Syllides* Ørsted, 1845

**Table:** 

1	Blades of some compound chaetae with one or more long basal spines	**2**
–	Blades of all compound chaetae with short, uniform spines on margin	**4**
2	Longer blades of each parapodium with 2–3 long basal spines	***Syllides japonica* Imajima, 1966** (1)
–	Blades of some compound chaetae with single, long basal spine	**3**
3	Blades of medium length compound chaetae with a long basal spine. Tips of dorsal simple chaetae blunt	***Syllides bansei* Perkins, 1981** (1)
–	Blades of longest and second pairs of compound chaetae with a long basal spine. Tips of dorsal simple chaetae enlarged and rounded, with some minute spines dorsally	***Syllides benedicti* Banse, 1971** (1)
4	Shafts of compound chaetae distally with 1–2 spines distinctly long and thick. Tips of dorsal simple chaetae enlarged and rounded	***Syllides convoluta* Webstery Benedict, 1884** (1)
–	Distal part of shafts with few, thin spines or smooth. Dorsal simple chaetae ending in a blunt tip	***Syllides fulva* (Marion & Bobretzky, 1875)** (1)

*Syllides longocirrata* Ørsted, 1845 is the type-species of the genus but it is poorly known. Later descriptions and reports of this species actually belong to a recently described species of another genus (*Streptospinigera* Kudenov, 1983) (Olivier et al. 2013).

### Key to genera Autolytinae Langerhans, 1879

**Table:** 

1	Dorsal cirri absent on some chaetigers	**2**
–	Dorsal cirri on all chaetigers	**3**
2	Antennae, tentacular cirri and dorsal cirri present on chaetiger 1; appendages absent on other chaetigers. Both simple and compound chaetae present	***Procerastea***
–	Dorsal cirri absent on chaetigers 2–5. Cirrostyles foliacious. All chaetae simple	***Imajimaea***
3	Large, clavate, dorsal cirri alternate with much smaller, cylindrical or clavate cirri. Nuchal epaulettes on special outgrowths	***Virchowia***
–	Not as above	**4**
4	Reproduction by epigamy	***Epigamia***
–	Reproduction by schizogamy	**5**
5	Trepan in two rows. Bayonet chaetae distally thick. Reproduction by anterior scissiparity	***Proceraea***
–	Trepan in single row. Bayonet chaetae distally slender. Reproduction by gemmiparity or anterior scissiparity	***Myrianida***

#### Genus *Procerastea* Langerhans, 1884

**Table:** 

1	Antennae, tentacular and dorsal cirri club-shaped. Trepan with 16–28 teeth. Chaetigers 1–4 with both unidentate and bidentate chaetae	***Procerastea halleziana* Malaquin, 1893** (1, 5)
–	Antennae, tentacular and dorsal cirri cylindrical. Trepan with 6–10 teeth. Chaetigers 1–4 with bidentate chaetae only	***Procerastea nematodes* Langerhans, 1884** (1, 5)

#### Genus *Virchowia* Langerhans, 1879

*Virchowia clavata* Langerhans, 1879 (1, 5)

#### Genus *Imajimaea* Nygren, 2004

*Imajimaea draculai* (San Martín & López, 2002) (1, 5, 16)

#### Genus *Epigamia* Nygren, 2004

**Table:** 

1	Trepan with two sizes of teeth, alternating between 1 large and 3–4 much smaller. Blades of compound chaetae with both teeth similar	***Epigamia alexandri* (Malmgren, 1867)** (5)
–	Trepan with three sizes of teeth, alternating between 1 large with 2 of medium size, or between 1 large, 1 small and 1 medium. Blades of compound chaetae with proximal tooth distinctly longer than distal	***Epigamia labordai* (San Martín & López, 2002)** (1, 5)

#### Genus *Proceraea* Ehlers, 1864

**Table:** 

1	Body without colour pattern	**2**
–	Body with colour pattern	**3**
2	Blades of compound chaetae with both teeth similar	***Proceraea aurantiaca* Claparède, 1868** (5)
–	Blades of compound chaetae with both teeth distinctly different; distal tooth smaller than proximal tooth	***Proceraea cornuta* (Agassiz, 1862)** (5)
3	Colour pattern of 3 lines	***Proceraea prismatica* (O. F. Müller, 1776)** (5)
–	Colour pattern otherwise	**4**
4	Colour pattern of 2 lines and brown squares	***Proceraea picta* Ehlers, 1864** (5)
–	Dorsum yellow with 2 black longitudinal lines on each side	***Proceraea scapularis* (Claparède, 1864)** (5)

#### Genus *Myrianida* Milne Edwards 1845

**Table:** 

1	Dorsal cirri distinctly flattened	***Myrianida pinnigera* (Montagu, 1808)** (1, 5)
–	Dorsal cirri cylindrical	**2**
2	Pharynx with several sinuations	**3**
–	Pharynx with 1–2 sinuations	**4**
3	Cirrophores swollen; cirrostyles attached subterminally on cirrophores. Trepan with indistinct teeth	***Myrianida inermis* (Saint-Joseph, 1886)** (5)
–	Cirrophores not swollen; cirrostyles attached terminally on cirrophores. Trepan with 9 distinct teeth	***Myrianida convoluta* (Cognetti, 1953)** (1, 5)
4	Cirrophores on both short and long cirri longer than cirrostyles in median chaetigers	***Myrianida sanmartini* Dietrich, Hager, Bönsch, Winkelman, Schmidt & Nygren**, in press (17)
–	Cirrophores on at least short cirri shorter than cirrostyles in all chaetigers	**5**
5	Teeth of trepan unequal	**6**
–	Teeth of trepan all of equal size	**8**
6	Colour pattern of 4 red spots on each segment. Trepan with 30–35 unequal teeth, 4–5 large and 26–30 small	***Myrianida rubropunctata* (Grube, 1860)** (5)
–	Not as above	**7**
7	Trepan with 22–29 teeth, alternating 1 large and 1-3 short	***Myrianida brachycephala* (Marenzeller, 1874)** (1, 5)
–	Trepan with 4–5 large teeth and 25–39 short	***Myrianida langerhansi* (Gidholm, 1967)** (5)
8–	Trepan with 12–24 teeth	***Myrianida quinquedecimdentata* (Langerhans, 1884)** (1, 5)
–	Trepan with 24–34 teeth	**9**
9	Cirri with more or less distinct alternation in length along body. Cirrophores on long cirri slightly longer than parapodial lobes	***Myrianida prolifera* (O. F. Müller, 1788)** (1, 5)
–	Cirri similar along body. Cirrophores on long cirri equal to parapodial lobes	***Myrianida edwardsi* (Saint-Joseph, 1886)** (1, 5)

### Keys to genera of Exogoninae Langerhans, 1879

Key based on reproductive and morphological characters

**Table:** 

1	Females brooding dorsally	**2**
–	Females brooding ventrally, developing juveniles, or viviparous	**4**
2	Two pairs of tentacular cirri. Body smooth	***Salvatoria***
–	Single pair of tentacular cirri. Body with papillae	**3**
3	Some dorsal cirri with a retractile cirrostyle. Antennae short. Pharynx relatively long and wide; pharyngeal tooth usually located far from anterior margin. Compound chaetae always with short, unidentate blades	***Prosphaerosyllis***
–	Antennae and dorsal cirri more or less elongate, without distal cirrostyle. Pharynx relatively slender; pharyngeal tooth usually located near anterior margin. Compound chaetae with elongate blades, bidentate, unidentate, or both	***Erinaceusyllis***
4	Body smooth	**5**
–	Body covered with papillae	***Sphaerosyllis***
5	Two pairs of tentacular cirri	***Brania***
–	Single pair of tentacular cirri	**6**
6	Palps basally fused to half or 2/3 of their length. Dorsal cirri bowling-pin shaped. Distinct parapodial glands	***Parapionosyllis***
–	Palps fused along their entire length or with terminal notch. Dorsal cirri small, papilliform. Parapodial glands indistinct or minute, apparently absent	**7**
7	Compound chaetae all bidentate falcigers, with both teeth similar; some species may have elongate, spiniger-like blades on some chaetae but their structure is similar to that of the shorter falcigers	***Parexogone***
–	Blades of compound chaetae of 2 different types; some elongated, spiniger-like, others short falcigers; some with blades missing or fused to shafts	***Exogone***

#### Key based exclusively on morphological features

**Table:** 

1	Two pairs of tentacular cirri	**2**
–	Single pair of tentacular cirri	**3**
2	Palps basally fused to half or 2/3 of their length. Dorsal cirri bowling-pin shaped or truncate. Parapodial glands distinct, sometimes inside dorsal cirri. Aciculae distally rounded, apparently hollow at tip. Pharynx slender, with distal soft papillae. Pharyngeal tooth conical, located at opening	***Brania***
–	Palps joined along most or all of their length by a dorsal membrane. Dorsal cirri spindle-shaped, usually elongate. Parapodial glands absent. Aciculae acuminate. Pharynx long and wide; usually without papillae on pharyngeal opening. Pharyngeal tooth rhomboidal to ovate, usually located far from pharyngeal opening	***Salvatoria***
3	Body without papillae	**4**
–	Body papillated	**6**
4	Palps basally fused to half or 2/3 of their length. Dorsal cirri bowling-pin shaped. Parapodial glands distinct. Dorsal simple chaetae distally serrated	***Parapionosyllis***
–	Palps fused along their entire length or with a distal, short notch. Dorsal cirri small, papilliform. Parapodial glands indistinct. Dorsal simple chaetae not as above	**5**
5	Compound chaetae all bidentate falcigers, with both teeth similar; some species may have elongate, spiniger-like blades on some chaetae but their structure is similar to that of the shorter falcigers	***Parexogone***
–	Blades of compound chaetae of 2 different types; some elongated, spiniger-like, others short falcigers; some with blades missing or fused to shafts	***Exogone***
6	Prostomium with 4 eyes, no additional eyespots. Proventriculus short, with few large muscular bands. Pharynx slender; pharyngeal tooth small, conical, located on anterior rim of pharynx. Aciculae with tip forming an angle (bulbous in one species)	***Sphaerosyllis***
–	Four eyes and 2 anterior eyespots on prostomium (sometimes difficult to see). Proventriculus barrel-shaped, long and relatively wide, with numerous, slender muscular bands. Pharynx relatively wide. Aciculae acuminate	**7**
7	Pharynx distinctly wide, without papillae. Pharyngeal tooth rhomboidal to oval, long, usually located far from anterior rim. Antennae and dorsal cirri typically having a retractile cirrostyle. Compound chaetae always with short, unidentate falcigers	***Prosphaerosyllis***
–	Pharynx proportionally more slender, sometimes with soft papillae surrounding opening. Pharyngeal tooth small, located near anterior rim. Antennae and dorsal cirri always without retractile cirrostyle. Compound chaetae usually with elongate blades bidentate, unidentate or both	***Erinaceusyllis***

#### Genus *Salvatoria* McIntosh, 1885

**Table:** 

1	Dorsal cirri short, absent from chaetiger 2	***Salvatoria swedmarki* (Gidholm, 1962)** (1)
–	Dorsal cirri elongated, present on all chaetigers	**2**
2	Blades of compound chaetae smooth on margin, unidentate or with a minute subdistal spine; 1–2 compound chaetae on each parapodium with longer blades having some long basal spines	***Salvatoria limbata* (Claparède, 1868)** (1)
–	Compound chaetae with bidentate blades	***Salvatoria clavata* (Claparède, 1863** (1)

#### Genus *Prosphaerosyllis* San Martín, 1984

**Table:** 

1	Antennae, tentacular and dorsal cirri minute, papilliform	***Prosphaerosyllis giandoi* (Somaschini & San Martín, 1994)** (6)
–	Antennae, tentacular and dorsal cirri typical of the genus, with a papilliform cirrostyle and a bulbous cirrophore	**2**
2	Blades of compound chaetae all short; dorsal ones with long spines, ventral ones smooth or very slightly spinulose	***Prosphaerosyllis campoyi* (San Martín, Acero, Contonente & Gómez, 1982)** (1)
–	Blades of compound chaetae without long spines	**3**
3	Dorsal papillae of two lengths, arranged in four longitudinal rows	***Prosphaerosyllis tetralix* (Eliason, 1920)** (1)
–	Dorsal papillae all similar, not arranged in longitudinal rows	**4**
4	Palps densely papillated. Dorsal papillae small, rounded. Without long papillae on dorsal cirri	***Prosphaerosyllis laubieri* Olivier, Grant, San Martín, Archambault & McKindsey, 2011** (7)
–	Palps with few papillae. Dorsal papillae digitiform	**5**
5	One long, distinct papilla on dorsal cirrus	***Prosphaerosyllis chauseyensis* Olivier, Grant, San Martín, Archambault & McKindsey, 2011** (7)
–	Without papillae on dorsal cirri	***Prosphaerosyllis xarifae* (Hartmann-Schröder, 1960)** (1)

#### Genus *Erinaceusyllis* San Martín, 2005

**Table:** 

–	Blades of compound chaetae unidentate	***Erinaceusyllis erinaceus* (Claparède, 1863)** (8) (19)
–	Chaetal blades bidentate	***Erinaceusyllis cryptica* (Ben-Eliahu, 1977)** (1)

#### Genus *Sphaerosyllis* Claparède, 1863

**Table:** 

1	Aciculae straight, with a bulbous distal swelling. Mid body parapodia with simple chaetae by loss of blades and shaft enlargement	***Sphaerosyllis bulbosa* Southern, 1914** (1)
–	Aciculae distally bent at an angle. Without enlarged chaetae	**2**
2	Antennae, tentacular and dorsal cirri minute, bulbous. Blades of mid body and posterior compound chaetae with smooth margins, with a long subdistal spine	***Sphaerosyllis parabulbosa* San Martín & López, 2002** (1)
–	Antennae, tentacular and dorsal cirri not so small, with longer tips. Blades otherwise	**3**
3	Without parapodial glands	**4**
–	With parapodial glands from chaetiger 4	**5**
4	Proventriculus rectangular. Compound chaetae of posterior parapodia with short, hooked, smooth blades	***Sphaerosyllis pirifera* Claparède, 1868** (1)
–	Proventriculus almost square. Compound chaetae with blades elongated throughout body	***Shaerosyllis* sp.**
5	Parapodial glands with granular material	***Sphaerosyllis glandulata* Perkins, 1981**(1) (*)
–	Parapodial glands with fibrillar material (rods)	**6**
6	Blades of compound chaetae with distinct dorsoventral gradation in length, especially on anterior parapodia	***Sphaerosyllis hystrix* Claparède, 1863** (1)
–	Blades of compound chaetae with, at most, very slight dorsoventral gradation in length; all blades short, those of dorsal compound chaetae with long spines on margin	***Sphaerosyllis taylori* Perkins, 1981** (1) (*)

(*) Stained specimens of species with fibrillar material can appear as *Sphaerosyllis glandulata*; parapodial glands with granular material are small, rounded and sometimes difficult to see; those with fibrillar material are ovate, large and easy to see.

#### Genus *Brania* Quatrefages, 1865

**Table:** 

–	Dorsal cirri truncate, with inclusions of fibrillar material	***Brania pusilla* (Dujardin, 1851)** (1)
–	Dorsal cirri bowling pin-shaped, with glands on parapodial bases	***Brania arminii* Langerhans, 1881**(1)

#### Genus *Parapionosyllis* Fauvel, 1923

**Table:** 

1	Compound chaetae with long blades; longer blades on each parapodium more than 3 times as long as shorter ones	**2**
–	Chaetae with shorter blades	**3**
2	Peristomium with a swelling partially covering the prostomium. Spines on long blades of compound chaetae short and straight. Two kinds of parapodial glands	***Parapionosyllis brevicirra* Day, 1954** (1)
–	Without swelling. Spines on long blades moderately long, distally dressed. Parapodial glands one kind, all with granular material	***Parapionosyllis macaronesiensis* Brito, Núñez & San Martín, 2000** (15)
3	Blades of uppermost compound chaetae in each parapodium twice as long as those of shortest chaetae, with long spines on margin	***Parapionosyllis elegans* (Pierantoni, 1903)** (1)
–	Blades of uppermost compound chaetae on each parapodium more than twice as long as those of shortest chaetae, without long spines	**4**
4	Blades of uppermost compound chaetae distinctly longer than others on each parapodium, about 3 times longer than of the most ventral.	***Parapionosyllis minuta* (Pierantoni, 1903)** (1)
–	Blades of uppermost compound chaetae longer than other blades on each parapodium but with a gradual and homogeneous gradation in size	***Parapionosyllis cabezali* Parapar, San Martín & Moreira, 2000** (1)

#### Genus *Parexogone* Mesnil & Caullery, 1918

**Table:** 

1	Compound chaetae of all parapodia with short blades, all similar or with slight dorsal to ventral gradation	***Parexogone hebes* (Webster & Benedict, 1884)** (1, 8)
–	Some compound chaetae (1–3) with long blades, at least on anterior parapodia	**2**
2	Dorsal simple chaetae with few (1–3) very long, thin spines (aristae), extending beyond the tips	***Parexogone longicirris* (Webster & Benedict, 1887)** (2)
–	Dorsal simple chaetae without aristae	**3**
3	All blades of compound chaetae elongated, slender, unidentate, with long, thin spines on margin. Aciculae with thin tips	***Parexogone campoyi* San Martín, Ceberio & Aguirrezabalaga, 1996** (1)
–	Most compound chaetae with short blades, without long spines on margin. Aciculae rounded distally	**4**
4	Lateral antennae minute; median antenna shorter than prostomium and palps combined	***Parexogone caribensis* San Martín, 1991** (1)
–	Lateral antennae similar in length to prostomium; median antenna longer than prostomium and palps combined	***Parexogone convoluta* (Campoy, 1982)** (1)

#### Genus *Exogone* Ørsted, 1845

**Table:** 

1	Spiniger-like compound chaetae modified, with enlarged, spinous shafts and short, triangular blades	***Exogone mompasensis* Martínez, Adarraga & San Martín, 2002** (1)
–	Chaetae not modified	**2**
2	Simple chaetae and blades of compound chaetae with long, thin spines extending beyond tips	***Exogone sorbei* San Martín, Ceberio & Aguirrezabalaga, 1996** (1)
–	Simple chaetae without these spines	**3**
3	Blades of falcigers with some long spines, extending beyond distal tooth	***Exogone lopezi* San Martín, Ceberio & Aguirrezabalaga, 1996** (1)
–	Without long spines on falcigers	**4**
4	Compound chaetae of 2–3 most anterior parapodia with blades very different from the others: very short, unidentate with a long basal spine	***Exogone naidina* Ørsted, 1845** (1)
–	Compound chaetae similar throughout	**5**
5	Median antenna distinctly longer than lateral antennae	***Exogone dispar* (Webster, 1879)** (1)
–	Median antenna small, similar to lateral antennae	***Exogone verugera* (Claparède, 1868)** (1)

### Key to genera of Syllinae Grube, 1850

**Table:** 

1	All chaetae simple, usually thick	***Haplosyllis***
–	Compound and capillary chaetae present dorsally and ventrally (sometimes some chaetae in mid body appear simple by blade and shaft fusion but typical compound chaetae also present anteriorly)	**2**
2	Body small, dorso-ventrally flattened. Antennae, tentacular and dorsal cirri reduced to a single, spherical article	**3**
–	Body of medium to large size, cylindrical or flattened. Antennae, tentacular and dorsal cirri with several articles (moniliform)	**4**
3	Palps fused. Two dorsal rows of spherical tubercles, similar to dorsal cirri	***Eurysyllis***
–	Palps separated. Without dorsal tubercles	***Plakosyllis***
4	Body cylindrical	***Syllis***
–	Body dorso-ventrally flattened	**5**
5	Dorsum, as well as antennae and dorsal cirri, with papillae and longitudinal grooves. Pharynx unarmed	***Xenosyllis***
–	Without longitudinal grooves on dorsum (minute transverse rows of papillae, difficult to see) (one species densely papillated). Pharynx with a trepan and, occasionally, a tooth	***Trypanosyllis***

#### Genus *Haplosyllis* Langerhans, 1879

*Haplosyllis spongicola* (Grube, 1855) (9)

#### Genus *Eurysyllis* Ehlers, 1864

**Table:** 

1	Compound chaetae with blades short and curved, smooth or with short spines on margin	***Eurysyllis tuberculata* Ehlers, 1864** (1)
–	Compound chaetae with blades elongated, with long spines on margin of anterior chaetae	***Eurysyllis mercuryi* Lucas, San Martín & Parapar, 2012** (10)

#### Genus *Plakosyllis* Hartmann-Schröder, 1956

*Plakosyllis brevipes* Hartmann-Schröder, 1956 (1)

#### Genus *Syllis* Lamarck, 1818

**Table:** 

1	Thick simple, Y-shaped chaetae in mid body (enlargement and fusion of shafts and blades)	***Syllis gracilis* Grube, 1840** (1)
–	Without these thick simple chaetae	**2**
2	Aciculae of posterior parapodia distally rounded and hollow. Pharyngeal tooth distinctly back from the pharyngeal opening	**3**
–	Aciculae not as above. Pharyngeal tooth located on anterior margin	**4**
3	Compound chaetae distinctly bidentate, with both teeth similar	***Syllis prolifera* Krohn, 1852** (1)
–	Compound chaetae with unidentate blades or with minute, spine-like proximal tooth	***Syllis vivipara* Krohn, 1869** (1)
4	With spiniger-like compound chaetae	**5**
–	Without spiniger-like compound chaetae	**12**
5	Aciculae of posterior parapodia thick, straight, acute, protruding from the parapodial lobes	**6**
–	Aciculae otherwise	**7**
6	Mid body dorsal cirri elongated. Mid body spiniger-like chaetae distinctly bidentate	***Syllis cornuta* Rathke, 1843** (11)
–	Mid body dorsal cirri fusiform. Mid body spiniger-like chaetae indistinctly bidentate	***Syllis mercedesae* Lucas, San Martín & Parapar, 2012** (10)
7	Proximal tooth of spiniger-like chaetae and falcigers distinct, forming a narrow angle with distal teeth (both teeth almost parallel); apparently without eyes	***Syllis caeca* (Katzmann, 1973)** (11)
–	Chaetae not as above; eyes present	**8**
8	Mid body dorsal cirri thick, short and fusiform	***Syllis parapari* San Martín & López, 2000** (1)
–	Dorsal cirri slender, more or less elongated	**9**
9	Posterior aciculae distally bent at an angle. Dorsal simple chaetae truncate. Short spiniger-like chaetae, distally rounded and unidentate from mid body	***Syllis rosea* (Langerhans, 1879)** (1, 11)
–	Aciculae acuminate. Dorsal simple chaetae acute. Arrangement and shape of spiniger-like chaetae not as above	**10**
10	Spiniger-like chaetae very short, only present on anterior and mid body segments; spiniger-like chaetae and falcigers unidentate, sometimes with a long, slender subdistal spine	***Syllis oerstedi* (Malmgren, 1867)**
–	Chaetae not as above	**11**
11	Blades of falcigers with long spines on margin, especially distally, extending beyond level of proximal tooth	***Syllis garciai* (Campoy, 1982)** (1)
–	Spines of blades not so long, decreasing distally, not reaching level of proximal tooth	***Syllis mauretanica* (Licher, 1999)** (11)
12	On mid body, one thick simple chaeta on each parapodium, formed by blade loss and shaft enlargement	***Syllis amica* Quatrefages, 1866** (1)
–	Without thickened, simple chaetae	**13**
13	Posterior aciculae distally bent at an angle. Dorsal simple chaetae truncate	**14**
–	Without the above characters	**15**
14	Proventriculus long, through about 5 segments or more. Two dorsal glands after proventriculus	***Syllis pulvinata* (Langerhans, 1881)** (1)
–	Short proventriculus, through 3 segments. Dorsal glands absent	***Syllis gerlachi* (Hartmann-Schröder, 1960)** (1)
15	Dorsal cirri of mid body short, fusiform	**14**
–	All dorsal cirri elongated, not fusiform	**16**
14	Dorsal cirri strongly fusiform. Mid body compound chaetae almost unidentate, with a short, small proximal tooth	***Syllis armillaris* (O.F. Müller, 1771)** (1, 11)
–	Dorsal cirri not so strongly fusiform. Mid body compound chaetae bidentate	***Syllis hyalina* Grube, 1863** (1, 11)
16	Aciculae of posterior parapodia thick, straight, acute, protruding from the parapodial lobes	**17**
–	Aciculae otherwise	**20**
17	Blades of compound chaetae unidentate (or slightly bidentate on anterior parapodia)	**18**
–	Blades distinctly bidentate	**19**
18	Dorsal cirri long. Blades distally more or less hooked	***Syllis fasciata* (Malmgren, 1867)** (11)
–	Dorsal cirri short, slender, delicate. Blades short, triangular	***Syllis licheri* Ravara, San Martín & Moreira, 2004** (1)
19	Dorsal cirri short, slender, delicate. Posterior aciculae distally bent, oblique, although pointed. Without colour pattern	***Syllis pontxioi* San Martín & López, 2000** (1)
–	Dorsal cirri longer. Aciculae straight. Strong pigmentation on anterior segments, as ∞	***Syllis variegata* Grube, 1860** (1, 11)
20	Compound chaetae all unidentate, distally acute	***Syllis vittata* Grube, 1840** (1, 11)
–	At least anterior compound chaetae bidentate	**21**
21	Long dorsal cirri of anterior segments distinctly thicker than others. Compound chaetae of posterior segments distinctly enlarged, unidentate or with a small proximal tooth. Anterior segments pigmented with distinct transverse red bands	***Syllis krohnii* Ehlers, 1864** (1, 11)
–	Dorsal cirri of similar thickness throughout body. Pigment pattern otherwise	**22**
22	Posterior compound chaetae unidentate by reduction and loss of distal tooth. Prostomium, peristomium and chaetiger 1 with dark red pigment, sometimes also a small red band on some anterior segments	***Syllis torquata* Marion & Bobretzky, 1875** (1)
–	Without such colour pattern nor such chaetae	**23**
23	Compound chaetae strongly bidentate. Colour pattern: one rhomboidal red mark on dorsum and a slight line on each border of each segment	***Syllis columbretensis* (Campoy, 1982)** (1)
–	Compound chaetae slightly bidentate Colour pattern forming ∞ on anterior segments	***Syllis westheidei* (San Martín, 1982)** (1, 11)

#### Genus *Xenosyllis* Marion & Bobretzky, 1875

*Xenosyllis scabra* (Ehlers, 1864) (1)

#### Genus *Trypanosyllis* Claparède, 1864

**Table:** 

1	Body densely papillated	***Trypanosyllis troll* Ramos, San Martín & Sikorski, 2010** (12)
–	Body non-papillated	**2**
2	Medium sized. Without colour pattern. Dorsal cirri short	***Trypanosyllis coeliaca* Claparède, 1868** (1)
–	Large. With colour pattern. Dorsal cirri long	**3**
3	Thin reddish transverse stripes on anteriormost segments. Some anterior dorsal cirri distinctly thicker and longer than others. Blades of compound chaetae slightly bidentate	***Trypanosyllis aeolis* Langerhans, 1879** (1)
–	Distinct colour pattern of transverse red stripes. Dorsal cirri long and red, all of similar thickness. Blades distinctly bidentate	***Trypanosyllis zebra* (Grube, 1860)** (1)

### Key to genera of Eusyllinae Malaquin, 1893 (and some “incertae sedis” genera)

**Table:** 

1	Pharyngeal tooth absent; pharynx with an incomplete trepan formed by few teeth, backwardly directed	***Odontosyllis***
–	Pharyngeal tooth present, with or without trepan	**2**
2	Pharynx with mid dorsal tooth and an incomplete arc of small denticles, frontally directed	**3**
–	Pharynx without denticles, only the mid dorsal tooth	**4**
3	All dorsal cirri long to very long, coiled over dorsum. Pharyngeal armature composed of a mid dorsal tooth and an incomplete arc of few (5–6) denticles	***Dioplosyllis***
–	Dorsal cirri not so long. Mid dorsal tooth and incomplete (sometimes complete) arc of numerous (around 30–40) pharyngeal denticles	***Eusyllis***
4	Antennae, tentacular cirri and dorsal cirri of chaetiger 1 long; subsequent dorsal cirri short	**5**
–	All appendages long	**6**
5	Body minute; strictly interstitial. Without enlarged, aciculiform ventral simple chaetae	***Neopetitia***
–	Body not so small; found on hard substrata. With enlarged, aciculiform, ventral simple chaetae	***Brevicirrosyllis***
6	Pharyngeal tooth on middle or posterior position or distinctly retarded	**7**
–	Pharyngeal tooth located on anterior margin	**8**
7	A number of anterior parapodia with enlarged aciculae, distally knobbed	***Streptodonta***
–	Without these enlarged aciculae	***Opisthodonta***
8	Segments posterior to proventriculus fused in units of 2–3 segments. Palps completely separated	***Synmerosyllis***
–	Segments not fused. Palps separated or basally fused	**9**
9	Without eyes (nuchal pigment patches may be present on prostomium). Palps long, fused to prostomium. Dorsal cirri of midbody short	***Palposyllis***
–	With eyes. Palps not so long nor fused to prostomium. Dorsal cirri long throughout body	**10**
10	Antennae and anterior dorsal cirri more or less articulated. A digitiform, subcirral papilla, below the bases of dorsal cirri	***Paraehlersia***
–	All appendages smooth. Without subcirral papillae	11
11	Small to minute size (< 5 mm in length). Palps separated. Pharynx shorter than proventriculus, with a long tooth. Compound chaetae unidentate or with small, spine-like proximal teeth	***Nudisyllis***
–	Medium to large size (> 5 mm in length). Palps fused basally. Pharynx similar in length or longer than proventriculus. Compound chaetae bidentate	***Pionosyllis***

#### Genus *Odontosyllis* Claparède, 1863

**Table:** 

1	Blades of compound chaetae elongated and unidentate	***Odontosyllis gibba* Claparède, 1863** (1)
–	Blades short and hooked, uni- or bidentate	**2**
2	Blades strongly bidentate	***Odontosyllis fulgurans* (Audouin & Milne Edwards, 1833)** (1)
–	Blades unidentate	***Odontosyllis ctenostoma* Claparède, 1868** (1)

#### Genus *Dioplosyllis* Gidholm, 1962

*Dioplosyllis cirrosa* Gidholm, 1962 (1)

#### Genus *Eusyllis* Malmgren, 1867

**Table:** 

1	Blades of compound chaetae all short and similar	***Eusyllis blomstrandi* Malmgren, 1867** (1, 8)
–	Compound chaetae with elongated and short blades on each parapodium	**2**
2	Ventral cirri of chaetiger 1 similar to remaining ones. Blades of compound chaetae of two distinctly different sizes. Aciculae thick, distally curved	***Eusyllis assimilis* Marenzeller, 1875** (1)
–	Ventral cirri of chaetiger 1 flattened, different from remaining ones. Blades of compound chaetae decreasing gradually in size on each parapodium. Aciculae slender, tricuspid	***Eusyllis lamelligera* Marion & Bobretzky, 1875** (1)

#### Genus *Neopetitia* San Martín, 2003

*Neopetitia amphophthalma* (Siewing, 1956) (1)

#### Genus *Brevicirrosyllis* San Martín, López & Aguado, 2009

*Brevicirrosyllis weismanni* (Langerhans, 1879) (1, 13)

#### Genus *Streptodonta* San Martín & Hutchings, 2006

**Table:** 

1	All chaetal blades short. Blades of compound chaetae and dorsal simple chaetae with a transluscent hood. Pharyngeal tooth located very far from anterior margin	***Streptodonta pterochaeta* (Southern, 1914)** (1)
–	Some chaetal blades distinctly longer than others. Chaetae without hood Pharyngeal tooth located more anteriorly	***Streptodonta exsulis* Ramos, San Martín & Sikorski, 2010** (12)

#### Genus *Opisthodonta* Langerhans, 1879

**Table:** 

1	Some blades of compound chaetae with proximal tooth curved, almost connecting with blade edge. Pharyngeal tooth on anterior 1/3 of pharynx	**2**
–	Proximal tooth not so curved. Pharyngeal tooth about half way along pharynx	***Opisthodonta morena* Langerhans, 1879** (1)
2	Blades of compound chaetae on mid body and posterior segments with distal tooth somewhat smaller than subdistal one	***Opisthodonta serratisetosa* López, San Martín & Jiménez, 1997** (1)
–	Distal tooth on blades minute or absent	***Opisthodonta longocirrata* (Saint-Joseph, 1886)** (1, 13)

#### Genus *Synmerosyllis* San Martín, López & Aguado, 2009

*Synmerosyllis lamelligera* (Saint-Joseph, 1886) (1, 13)

#### Genus *Palposyllis* Hartmann-Schröder, 1977

**Table:** 

1	Dorsal cirri absent from chaetiger 2. Palps distinctly long. Body with retractile papillae	***Palposyllis prosostoma* Hartmann-Schröder, 1977** (1)
–	Dorsal cirri present on chaetiger 2. Palps not so long. Without retractile papillae	***Palposyllis propeweismanni* (Dauvin & Lee, 1983), comb. n.** (14) (*)

(*) San Martín et al. (2009) considered this species as synonymous with *Palposyllis prosostoma*; however, after examination of new material during the NMBAQC Workshop, it seems to be a different species.

#### Genus *Paraehlersia* San Martín, 2003

**Table:** 

1	Blades of posterior compound chaetae short, with proximal tooth distinctly longer than distal tooth	***Paraehlersia ferrugina* (Langerhans, 1881)** (1)
–	Blades similar throughout, with proximal tooth shorter than distal	***Paraehlersia dionisi* Núñez & San Martín, 1991** (1, 13)

#### Genus *Nudisyllis* Knox & Cameron 1970

**Table:** 

1	Long blades of compound chaetae bidentate, with both teeth similar. Short blades unidentate	***Nudisyllis pulligera* (Krohn, 1852)** (1, 13)
–	All blades unidentate or with minute, spine-like subdistal tooth	***Nudisyllis divaricata* (Keferstein, 1862)** (1, 13)

#### Genus *Pionosyllis* Malmgren, 1867

**Table:** 

1	Small size (up to 10 mm long). Teeth of blades of compound chaetae close to each other	***Pionosyllis compacta* Malmgren, 1867** (13)
–	Large size (up to 31 mm long. Teeth of blades well separated	***Pionosyllis enigmatica* (Wesenberg-Lund, 1950)** (1, 13)
